# Tamoxifen Resistance: Emerging Molecular Targets

**DOI:** 10.3390/ijms17081357

**Published:** 2016-08-19

**Authors:** Milena Rondón-Lagos, Victoria E. Villegas, Nelson Rangel, Magda Carolina Sánchez, Peter G. Zaphiropoulos

**Affiliations:** 1 Department of Medical Sciences, University of Turin, Turin 10126, Italy; nrangel@unito.it; 2 Faculty of Natural Sciences and Mathematics, Universidad del Rosario, Bogotá 11001000, Colombia; magda.sanchez@urosario.edu.co; 3 Doctoral Program in Biomedical Sciences, Universidad del Rosario, Bogotá 11001000, Colombia; 4 Department of Biosciences and Nutrition, Karolinska Institutet, Huddinge 14183, Sweden; peter.zaphiropoulos@ki.se

**Keywords:** tamoxifen, breast cancer, G protein-coupled estrogen receptor (GPER), estrogen receptors (ERs), androgen receptor (AR), Hedgehog (HH) signaling pathway, endocrine resistance

## Abstract

17β-Estradiol (E2) plays a pivotal role in the development and progression of breast cancer. As a result, blockade of the E2 signal through either tamoxifen (TAM) or aromatase inhibitors is an important therapeutic strategy to treat or prevent estrogen receptor (ER) positive breast cancer. However, resistance to TAM is the major obstacle in endocrine therapy. This resistance occurs either de novo or is acquired after an initial beneficial response. The underlying mechanisms for TAM resistance are probably multifactorial and remain largely unknown. Considering that breast cancer is a very heterogeneous disease and patients respond differently to treatment, the molecular analysis of TAM’s biological activity could provide the necessary framework to understand the complex effects of this drug in target cells. Moreover, this could explain, at least in part, the development of resistance and indicate an optimal therapeutic option. This review highlights the implications of TAM in breast cancer as well as the role of receptors/signal pathways recently suggested to be involved in the development of TAM resistance. G protein—coupled estrogen receptor, Androgen Receptor and Hedgehog signaling pathways are emerging as novel therapeutic targets and prognostic indicators for breast cancer, based on their ability to mediate estrogenic signaling in ERα-positive or -negative breast cancer.

## 1. Introduction

Breast cancer is the most frequent type of cancer in women from developed and developing countries. It represents 23% of all female cancers, and often leads to death, even though the mortality rates are quite lower than the incidence rates [1]. However, breast cancer shows high morbidity and is commonly related to a wide variety of risk factors, including genetic predisposition and exposure to estrogens.

Prolonged exposure to estrogen represents a significant risk factor in the development of breast cancer; however, the mechanisms whereby estrogens enhance the incidence of breast cancer are not completely understood and the subject of a certain controversy. Estrogens could promote de novo breast cancer development though either receptor-dependent or -independent mechanisms [2]. It is known that estrogens bind to a specific nuclear receptor, the estrogen receptor (ER), which generates a potent stimulus for breast gland cell proliferation and increases the risk of DNA mutation during replication [3,4,5,6]. However, some studies in ER knockout mice resulted in a sufficiently high incidence of tumor development, indicating that estrogens can promote breast cancer through ER-independent mechanisms [7]. The G protein-coupled estrogen receptor (GPER) is one candidate for this non-ER signaling that is mediated by estrogens [8].

The therapeutic management of ERα-positive patients consists in the application of endocrine strategies that seek to block ER with anti-estrogens, such as tamoxifen (TAM) or in the depletion of ligand (estrogen) availability, either by suppressing the gonads in premenopausal women (ovariectomy) or by using aromatase inhibitors (AIs) in postmenopausal women. These strategies are implemented both for early and metastatic breast cancer. However, not all patients respond to TAM endocrine therapy, and moreover, patients that initially respond to treatment can acquire resistance to this drug [9,10,11,12].

Currently, the therapeutic management of ERα-positive breast tumors with acquired resistance to TAM consists in the application of second line therapies such as AIs [13], or the synthetic ER antagonist fulvestrant. AIs, including exemestane, letrozole and anastrozole, seek to disrupt estrogen signaling by either irreversible and inactivating binding (exemestane) or reversible and competitive binding (letrozole and anastrazole) to the aromatase enzyme; thus significantly reducing local estrogen biosynthesis and intratumoral levels of estrogen [14,15]. Fulvestrant prevents ER dimerization, leading to degradation and loss of cellular ER, and has proven to be as effective as anastrozole in treating postmenopausal women with acquired TAM resistance [16,17].

Additional approaches involve the use of agents designed to resensitize resistant tumors to endocrine therapy by targeting pathways recognized as drivers of resistance. One such approach has been the combination of TAM endocrine therapies with growth factor receptor kinase inhibitors (RKIs), such as gefitinib, trastuzumab and lapatinib [18,19]. The use of these combined therapies was shown to be a good therapeutic option to either prevent or overcome resistance to TAM in cancers overexpressing epidermal growth factor receptor (EGFR) or EGFR2 (HER2) [18,19,20,21].

To date, several studies have led to the identification of key factors/signaling pathways involved in TAM resistance. These include activation of ER signaling, overexpression/up-regulation of receptor tyrosine kinases (RTKs) signaling pathways (EGFR, HER2, insulin-like growth factor 1 receptor (IGF1R) and fibroblast growth factor receptor (FGFR)), deregulation of the PI3K-PTEN/AKT/mTOR pathway and hyperactivation of NF-κB signaling [22]. However, considering that breast cancer is a heterogeneous disease, multiple mechanisms may contribute to TAM resistance, highlighting the importance of identifying prognostic biomarkers that would allow the application of more effective therapeutic options.

Recently, additional receptors/pathways have been postulated to be involved in the development of TAM resistance. In this review, we highlight the role of these new receptors/pathways and their exploitation as both novel prognostication markers and therapeutic targets in breast cancer.

## 2. Estrogen Receptors (ERα and ERβ)

### 2.1. Structure

**Figure 1:**
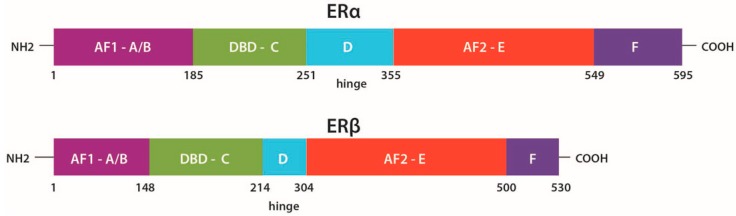
Functional domains of estrogen receptors (ERα and ERβ). Receptor domains are indicated in different colors: Purple, activation factor 1 (AF1) domains A/B; green, DNA-binding domain (DBD) C; blue, heat shock proteins binding domain D; red, activation factor 2 (AF2) domain E; dark purple, C-terminal domain F. Modified from Ng et al. [26].

ERs are highly involved in the development and progression of breast cancer. Most of the effects of 17β-Estradiol (E2) are mediated through its two nuclear receptors: ERα (ERα) and β (ERβ), which are encoded by different genes, *ESR1* encodes ERα on chromosome 6 and *ESR2* encodes ERβ on chromosome 14 [23,24]. ERβ is more abundant than ERα in normal human and mouse mammary gland [3,9,25] and both receptors contain in their structure different domains: Two ligand-independent transcriptional activation, N-terminal domains, NTD (A/B domains), also called activation factor 1 (AF1) domains, where MAPKs-mediated phosphorylation is carried out, a DNA-binding domain, DBD (C domain), a nuclear localization and heat shock proteins binding domain (domain D), a ligand-dependent transcriptional activation, ligand binding domain, LBD (domain E), also called activation factor 2 (AF2) domain and a C-terminal domain (domain F), which modulates the transcriptional activation mediated by domains A/B and E [3,26,27,28] (Figure 1).

In the absence of ligands, ERs are found predominantly in the nucleus as monomers associated with multiprotein complexes, including heat shock proteins (HSPs) [27,28]. However, recent studies have reported the presence of ERα, ERβ or both on the inner phase of the plasma membrane, bound either to membrane proteins, e.g., caveolin-1, or associated to other membrane receptors, e.g., Insulin-like growth factor receptor (IGFR), EGFR or HER2, or to signal adapter molecules, e.g., SHC (Src Homology 2 Domain Containing) [27,28,29].

### 2.2. Function

ERs belong to a family of nuclear proteins bound to DNA, which regulate the transcription of a wide variety of genes involved in the development and function of the reproductive organs, in bone density, in regulation of the cell cycle, in DNA replication, differentiation, apoptosis, angiogenesis, survival and tumor progression. Examples of genes regulated by ERs include *IGFR*, *cyclin D1* (*CCND1*), *B-cell CLL/lymphoma 2 (BCL-2)*, *vascular endothelial growth factor* (*VEGF*) and certain growth factors, e.g., heregulins (HER), transforming growth factor β (TGFβ) and amphiregulins, which bind and activate EGFR [28,30,31].

The classical mechanism of action of ERs (genomic signaling) initiates with E2 binding to ER receptors (α and β) in the nucleus and subsequent binding of the ERs to DNA in regulatory regions termed estrogen response elements—EREs. However, ERs can also regulate the expression of many genes without direct binding to DNA, through protein–protein interactions with transcription factors, f. ex. specificity protein 1 (Sp-1), activator protein 1 (AP-1) and GATA binding protein 1 (GATA1) [32,33,34,35]. Genes activated by this route include *IGF-1*, *c-MYC*, *CCND1*, *c-FOS* and the low-density lipoprotein receptor [36].

Besides this classical mechanism, a non-genomic effect mediated by membrane-associated ERα and ERβ has also been observed, leading to the activation of the cytoplasmic tyrosine kinase Src and other signaling molecules including: (i) IGF1R and EGFR; (ii) mitogen-activated protein kinases (MAPK), phosphatidyl inositol 3 kinase (PI3K) and AKT; (iii) protein kinase C (PKC) and cyclic AMP (cAMP); (iv) p21 and (v) pathways that promote the release of intracellular calcium [37,38,39,40,41]. These signaling cascades can phosphorylate nuclear ERs and their co-activators (AIB1/SRC-3) resulting in their activation as transcriptional regulators of target genes [42]. In addition, the G protein-coupled estrogen receptor (GPER) is another candidate molecule involved in the non-genomic signaling mediated by E2 [8] and also implicated in TAM resistance [8,42,43,44].

In normal breast tissue, ERβ plays a role as the dominant receptor, but during carcinogenesis the amount of ERβ decreases whilst the amount of ERα increases. Thus, ERβ was postulated to act as a tumor suppressor gene in breast cancer [45]. Most of the ERs present in breast tumors are ERα; moreover, high levels of this receptor in benign breast epithelium increase the risk to develop breast cancer, and ERα has particularly been associated with tumor initiation and progression to later stages. Although the detailed ERβ function in breast and ovarian cancer is still unclear, the interaction between ERβ and ERα is essential for normal development and for the functionality of the tissues in which they are expressed [28,31,34].

ER detection is widely used in patients with breast cancer as a prognostic marker to predict the risk of progression and as a response predictor to anti-estrogen therapy. Tumors positive for ER and progesterone receptor (PR) are very well differentiated in histological terms; they show low rates of cell proliferation and diploid content of DNA. Breast tumors can also be associated with poor prognostic markers, e.g., amplification of the *HER2*, *c-MYC* and *INT-2* genes and *TP53* gene mutation [46,47].

## 3. Tamoxifen (TAM)

### 3.1. Function

TAM is a non-steroidal anti-estrogen with mixed ER agonist/antagonist activities; its introduction represented a pioneering therapy for the treatment of ERα-positive breast cancer and since then has extensively been used. TAM’s activity is dependent on circulating E2 levels, which are higher in pre-menopausal women and lower in postmenopausal women. Initially considered an antagonist, TAM is currently classified as a Selective Estrogen Receptor Modulator (SERM), a compound that exhibits tissue-specific ER agonist or antagonist activity. It binds competitively to the ERs, thereby inhibiting E2 dependent gene transcription, cell proliferation and tumor growth [48,49,50].

**Figure 2:**
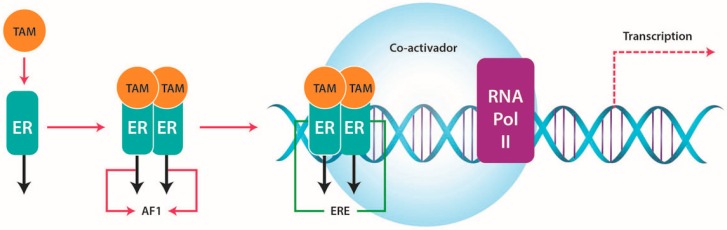
Tamoxifen action. The tamoxifen-estrogen receptor (TAM-ER) complex activates the activation factor 1 (AF1) domain and inhibits the activation factor 2 (AF2) domain. The TAM-ER dimer binds to DNA at estrogen response element (ERE) sequences in the promotor region of E2 responsive genes. Transcription of these genes is attenuated because the AF2 domain is inactive.

TAM binds to ER with lower affinity compared with E2, and dissociates the heat shock protein 90 (HSP90). The TAM-ER complex homo or hetero dimerizes and translocates to the cell nucleus, causing the activation of the activation factor 1 (AF1) domain and inhibiting the activation factor 2 (AF2) domain. Following this, the TAM-ER dimer binds to DNA at palindromic ERE sequences in the promoter region of E2 responsive genes. Transcription of the E2 responsive gene(s) is attenuated because the AF2, ligand-dependent domain is inactive, and ER co-activator binding is reduced by the TAM-ER complex; partial agonist activity results from the AF1 domain, which remains active in the TAM-ER complex [51] (Figure 2).

**Figure 3:**
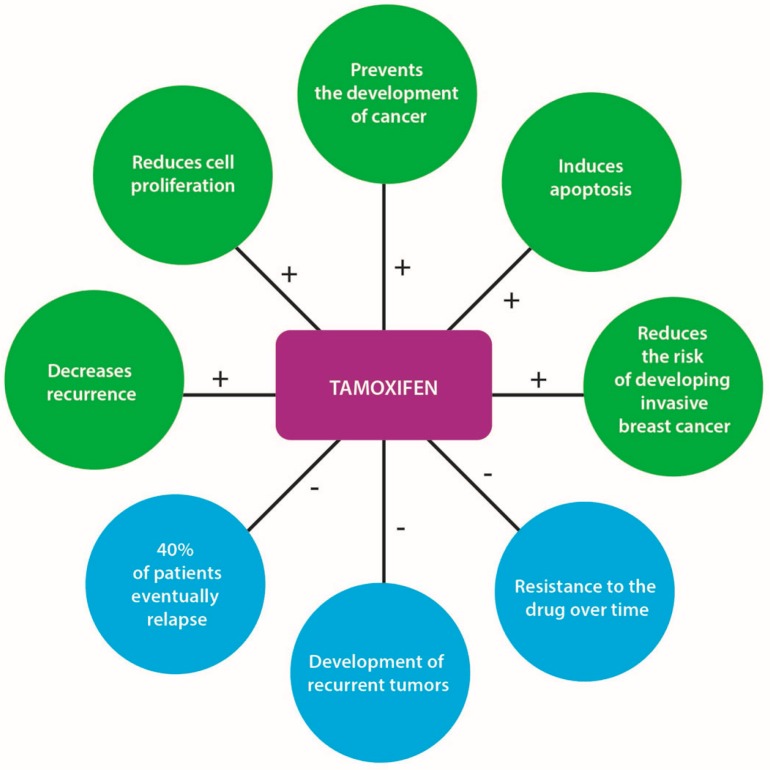
Tamoxifen’s positive and negative effects. Positive effects are indicated by a plus sign (+) and negative effects by a minus sign (−).

TAM was demonstrated to reduce the risk of developing ERα-positive breast cancer by at least 50%, in both pre- and post-menopausal women [52]. The use of this anti-estrogen agent (dose of 20 mg/day) reduces the occurrence of breast cancer by 38% in healthy women at high risk of acquiring the disease, decreases the likelihood of recurrence in early breast cancer cases, prevents the development of cancer in the opposite breast, reduces cell proliferation, induces apoptosis and reduces the risk of developing invasive breast cancer in women with Ductal Carcinoma In Situ (DCIS) [53,54] (Figure 3). Five years of TAM treatment prevents ERα-positive breast cancer not only during this time period but also after treatment cessation. Recent studies have found that extending the duration of TAM treatment to 10 years further reduces the risk of breast cancer recurrence in ERα-positive cases [52,55].

Some studies have suggested that TAM causes cell cycle arrest and apoptosis both in vitro and in vivo by modulating growth factors, e.g., down-regulation of TGFα, induction of stromal TGFβ1 and decrease in the production of the potent mitogen IGF-1. Other studies have showed that TAM inhibits cell proliferation by inducing cell cycle arrest in the G0/G1 phase. In addition, it has been reported that TAM can stimulate cellular proliferation by acting on several signaling pathways, including *c-MYC* and MAPKs. This mitogenic effect might arise as a result of estrogen-altered metabolism [56,57,58,59].

### 3.2. TAM Metabolism

**Figure 4:**
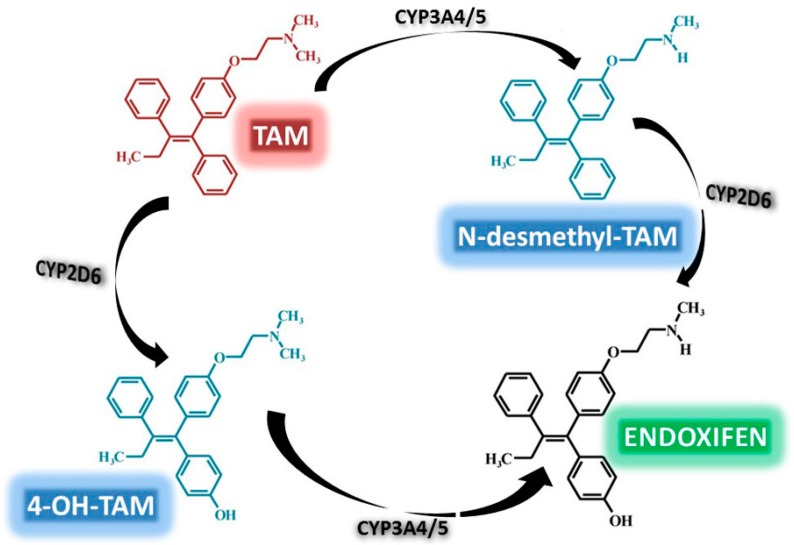
TAM metabolism. TAM is extensively metabolized through biochemical reactions mainly catalyzed by the cytochrome P450 family of enzymes (CYP3A4/5, CYP2D6). Based on Stearns et al. [62].

TAM is extensively metabolized in the liver and to a lesser extent locally in the breast, with the main excretion occurring via the bile and the feces. Cytochrome P450 enzymes (CYP) mediate the biotransformation of TAM to several primary and secondary products, mainly through demethylation and hydroxylation. The major metabolic pathway involves initial conversion of TAM to *N*-desmethyl-TAM via CYP3A4/5, followed by conversion of *N*-desmethyl-TAM to endoxifen, via CYP2D6. In addition, some TAM is initially metabolized by CYP2D6 to the active metabolite 4-hydroxy-tamoxifen (4-OH-TAM), which in turn is either degraded or converted by CYP3A4/5 to endoxifen [60,61,62] (Figure 4). Endoxifen and 4-OH-TAM have higher potencies than the parental compound and it has been suggested that these metabolites may be responsible for the anti-tumor effects of TAM in vivo [63,64].

TAM and its metabolites bind to the ERs, albeit with somewhat different affinities. They block the binding of E2 to these receptors, prevent the conformational changes required for binding of co-activators and lead to the preferential recruitment of co-repressors, including nuclear receptor corepressor 1 (NCOR1). The reduced transcriptional activity of the ERs results in attenuation of tumor growth, as the genes regulated by E2 are involved in proliferation, angiogenesis and anti-apoptosis [61,64].

Endoxifen, the major metabolite responsible for the action of TAM in vivo, appears to have differential effects on the two ER receptors. It stabilizes ERβ, promoting receptor hetero-dimerization and has increased inhibitory effects on the expression of target genes. On the other hand, endoxifen targets ERα for proteasomal degradation in breast cancer cells. Polymorphisms in several CYP enzymes involved in TAM metabolism impact on the relative abundance and availability of the metabolites and, consequently, on their effects in E2-dependent breast cancer cell proliferation [63,65,66].

## 4. TAM Resistance

Despite the obvious benefits of TAM in patients at all stages of ERα-positive breast cancer, several studies have reported that the tumors in almost all patients with metastatic disease and in 40% of patients receiving TAM as adjuvant therapy eventually relapse, with a deadly outcome. Likewise, post-menopausal women with early stage breast cancer that initially responded well to TAM can develop recurrent tumors not only in the breast, but also in the endometrium [31], and over time become resistant to the drug [67].

Several mechanisms are proposed to explain TAM resistance, and intensive research has resulted in the identification of molecular pathways that may be involved. These include ER signaling, RTKs signal transduction pathways (HER2, EGFR, FGFR, and IGF1R), the phosphatidylinositol 3-kinase-phosphatase and tensin homolog (PI3K-PTEN)/V-Akt murine thymoma viral oncogene homolog (AKT)/mechanistic target of rapamycin (mTOR) pathway and NF-κB signaling [11,12,22,68].

Additional mechanisms for TAM resistance implicate imbalances between E2 anabolism and catabolism [69], altered bioavailability of TAM [70], increased angiogenesis, heterogeneity of tumor cell population or overexpression of growth factors. In fact, experimental evidence suggests that patients over-expressing the HER2 protein can develop resistance to TAM [50,71,72], however, the mechanism by which this occurs is unknown.

### 4.1. ERs and TAM Resistance

ERα is expressed in 70%–80% of breast tumors and ERα-positivity is a well-established predictor of a good response to TAM treatment. Patients with higher levels of ERα (ERα-positive tumors) show increased benefits to TAM therapy compared to patients with lower receptor expression [73,74]. Patients with ERα-negative tumors are considered as no responders, although 5%–10% of these do benefit from adjuvant TAM therapy [74,75,76,77]. The response to TAM is frequently limited in duration because the patients can develop resistance [31,32,67,78], with this being one of the major problems of endocrine therapy. The possible causes of resistance to TAM that implicate the ERs are:

#### 4.1.1. Increased Growth Factor Signaling and Membrane-Associated ERs

The non-genomic activities of membrane-associated ERs facilitate cross-communication between these receptors and RTKs signaling pathways, including HER2, EGFR, and the PI3K pathway. The membrane ERs can activate RTKs signaling and, in turn, these can phosphorylate ERα at Ser 118 or Ser 167 within the AF1 domain by MAPK and AKT, respectively, which are downstream components of the EGFR/HER2 signaling pathway. This interaction leads to ligand-independent activation of ER and to increased cellular proliferation [12]. This effect is not relevant for breast cancer cells with low levels of membrane ERs and in which RTKs, such as HER2, are poorly expressed [79].

Furthermore, in ERα-positive/HER2-positive tumors, TAM apparently acts as an E2 agonist contributing to increased cell proliferation and survival. In addition, it has also been reported that TAM can activate the membrane-associated ERs in a manner analogous to E2 ligands, thus accounting for its agonistic effects and the observed cellular resistance to this compound [79,80]. Considering the above, cross-communication between ERs and RTKs signaling pathways can contribute to TAM resistance and promote the survival of breast cancer cells [32,35,78]. In fact, the potential involvement of this cross-communication has also been observed in a meta-analysis, in which the tumors of patients with metastatic ERα-positive/HER2-positive breast cancer relapsed after a short period of TAM treatment compared to patients with HER2-negative cancers [81]. Overexpression and activation of EGFR and HER2 lead to proliferation and cell survival through activation of MAPK and PI3K/AKT signaling pathways, thus contributing to the development of resistance to endocrine therapy [28,35].

#### 4.1.2. Loss of ERα Expression

It has been reported that another cause of resistance to TAM is the loss of expression of ERα Since the effects of TAM are primarily mediated through the ERα, and the degree of ERα expression is a strong predictor of a positive response to TAM, loss of ERα expression may be the main mechanism of de novo resistance to endocrine therapy. Loss of ERα expression has mainly been associated with aberrant methylation of CpG islands and with increased deacetylation of histones, which result in a more compact nucleosome structure that limits transcription [82,83]. Additionally, loss of ERα expression has also been linked with invasiveness and poor prognosis [84].

Moreover, it has been hypothesized that loss of ERα expression might be responsible for acquired resistance to TAM; however, some studies have reported that only 17%–28% of tumors with acquired resistance to TAM do not express ERα [85,86], and approximately 20% of TAM-resistant tumors will eventually respond to a second-line of treatment with AIs or fulvestrant [87,88].

#### 4.1.3. ERα Mutations

In hyperplastic breast lesions, a single amino-acid substitution in ERα, which leads to a change at position 303 from lysine to arginine has been detected (K303R). This mutation produces a receptor with enhanced properties in ER-mediated cell growth, as it has increased sensitivity to estrogen and altered crosstalk with various cellular pathways that normally down-regulate ER signaling. These changes of ER activity could contribute to the development of endocrine resistance, but no clinical evidence supporting this claim has yet been reported [84]. In addition, the significance of this mutation is unclear, since it occurs at a low frequency (5%–10%) [89,90] and, moreover, has not been detected in large datasets, including the TCGA dataset [91,92].

Recently, several studies have reported the presence of mutations on the Ligand Binding Domain (LBD) of ERα in ERα-positive breast tumors, including mutations p.Tyr537Ser/Asn, p.Asp538Gly [93,94,95,96] and p.Leu536Gln [97]. These mutations promote the agonist conformation of ERα in the absence of ligand, thus leading to hormone-independent tumor cell growth and clinical resistance to hormonal therapy [93,94,95,97]. The reported incidence on these mutations was low (less than 1%) in primary tumors but high (11%–55%) in metastatic ERα-positive breast cancer [93,96,97]. Therefore, these mutations occur almost exclusively in metastatic breast tumors [96]. Interestingly, ERα mutations appear to be frequently acquired in patients who previously have received hormonal treatment [96].

### 4.2. (G-Protein Coupled Estrogen Receptor) GPER and TAM Resistance

GPER, formerly known as GPR30, is a candidate molecule that can mediate non-genomic E2 signaling [8] and also may have a role in TAM resistance. GPER, a seven transmembrane domain protein, has recently been identified as a novel estrogen receptor structurally distinct from the classic ERs (ERα and ERβ) [64,98]. This protein is expressed in approximately 50%–60% of all breast carcinomas [64,99], in endometrial and ovarian cancer cells, in thyroid carcinoma cell lines [29], in ERα-positive (MCF7), ERα-negative (SKBR3) and triple negative breast cancer (TNBC) cells [42].

#### 4.2.1. GPER Subcellular Localization

Although the subcellular localization of GPER is still a subject of debate, some studies have indicated that GPER is located mainly in the nucleus, however, this receptor has also been observed in the cytoplasm [100]. This differential subcellular localization could be explained by a retrograde transport of GPER from the plasma membrane towards the nucleus.

Interestingly, the subcellular localization of GPER has been associated with different clinicopathological characteristics. While cytoplasmic GPER localization is correlated with low tumor stage and ER and PR positive breast carcinomas, nuclear GPER is linked to poorly differentiated carcinomas and TNBC subtypes [100,101]. These observations reflect the differential biological significance of the two subcellular distributions, one associated with a better clinical outcome (cytoplasmic GPER) and the other with less favorable tumor prognosis (nuclear GPER).

#### 4.2.2. GPER Signaling

Signaling through GPER occurs via transactivation of EGFR and involves tyrosine kinases of the Src family. In this mechanism, E2 initially binds to GPER eliciting the activation of heterotrimeric G protein–tyrosine kinase Src-matrix metalloproteinase signaling [64], leading to the production of heparin-binding epidermal growth factor (HB-EGF). HB-EGF binding to EGFR activates the mitogen-activated protein kinase/extracellular regulated protein kinase (MAPK/ERK) signaling cascade [64,102] and increases adenylate cyclase activity. The increased cyclic AMP (cAMP) levels promote the phosphorylation of the cAMP response element-binding (CREB) transcription factor, which subsequently binds to cAMP-response elements (CRE) on promoters of mitogenic genes [103].

**Figure 5:**
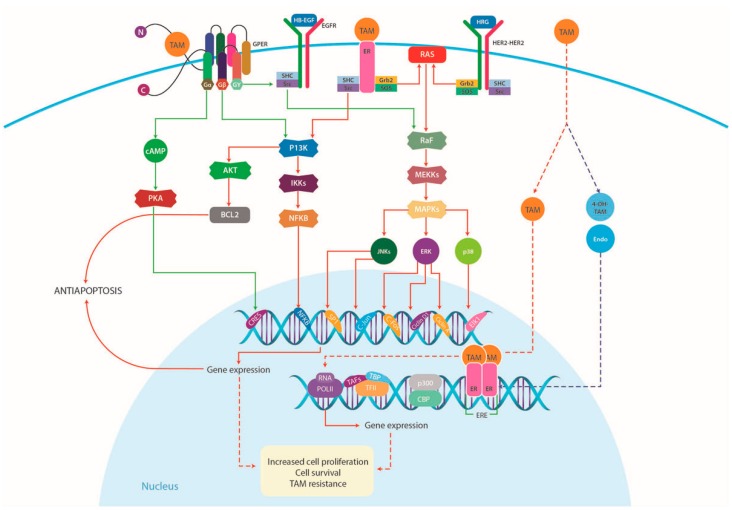
Proposed model of cell proliferation mediated by TAM in breast cancer cells. TAM can activate both GPER and membrane-associated ERs and crosstalk with growth factor signaling pathways, including HER2 and EGFR receptors, in a manner analogous to E2. TAM resistance can therefore be attributed, at least partly, to the GPER receptor, suggesting a role for GPER in non-classical steroid hormone actions. In fact, TAM as well as its metabolite, 4-OH-TAM, have high-affinity binding to GPER and mimic the rapid, non-genomic E2 effects in breast cancer cells. EGFR, epidermal growth factor receptor; ER, estrogen receptor; GPER, G-protein coupled estrogen receptor; HB-EGF, heparin-binding epidermal growth factor; HRG, heregulins; Src, cytoplasmic tyrosine kinase; MAPK, mitogen-activated protein kinases; PI3K/Akt, phosphatidylinositol 3-kinase/protein kinase B; PKC, protein kinase C; cAMP, cyclic AMP. Modified from SABiosciences (http://www.sabiosciences.com/pathway.php?sn=Estrogen_Pathway).

In addition to E2, TAM as well as its metabolite, 4-OH-TAM, have also high-affinity binding to GPER and can activate the receptor [104,105,106,107], thus inducing rapid cellular signaling, including ERK activation, PI3K activation, calcium mobilization and cAMP production in breast cancer cells [98,105] (Figure 5).

The identification of this distinct class of steroid receptors, i.e., GPER, suggests a role for GPER in non-classical steroid hormone actions [4,7,8,32,108,109]. Consequently, E2 and TAM are regarded as GPER agonists [64]. This activation of GPER signaling often causes tumor progression, which makes GPER a potential therapeutic target in breast cancer.

#### 4.2.3. GPER in Breast Cancer

Recent studies have provided evidence that high levels of GPER protein expression in breast cancer patients correlate with clinical and pathological biomarkers of poor outcome, including increased tumor size and metastasis [64,101,110]. Moreover, it has been reported that in GPER-positive patients, TAM activates the crosstalk between the GPER and the EGFR signaling pathways. GPER activation increased ligand-dependent EGFR activity, leading to an ERK1/2-mediated transcriptional response. This crosstalk elicits an increased cellular growth that is associated not only with TAM resistance (Figure 5), but also with metastasis [43,98]. In this regard, several studies have demonstrated that in patients with GPER-positive tumors, treatment with TAM increases GPER expression, with the overall survival decreasing in comparison to patients who did not receive TAM. These results suggest that in breast cancer patients with high GPER expression, potential treatment with TAM should be carefully evaluated [64,101]. In such cases, the capacity of GPER to mediate E2 action is significantly enhanced during the development of TAM resistance [43].

In addition to the above mechanisms, a recent study has shown that ligand-activated GPER also triggers NOTCH activation and expression of NOTCH target genes. Moreover, NOTCH signaling contributes to GPER-mediated migration in ER-negative breast cancer cells and cancer-associated fibroblasts [111].

#### 4.2.4. GPER in Triple Negative Breast Cancer (TNBC)

TNBC cancers, defined as tumors that lack ER, PR and HER2 expression, account for 12%–17% of all invasive breast carcinomas and comprise a heterogeneous group of tumors, with varying histological features and clinical behavior [112].

Recently, GPER has been proposed as a candidate biomarker for TNBC that has a role in growth regulation. Studies in TNBC patients revealed that these tumors strongly express GPER and that this expression correlates with higher TNBC recurrence [113]. While it is well known that ERα-positivity is a predictor of response to TAM, it is also clear that 5%–10% of ERα-negative patients do benefit from adjuvant TAM treatment [75,76,77,114]. In these cells, TAM enhances mRNA and protein expression of CCNA1 and CCND1 after 3 h and 12 h of treatment, indicating not only that TAM regulates cell cycle progression via GPER/EGFR/ERK signaling but also suggesting a link between estrogen, TAM and GPER in TNBC (Figure 5) [42]. Taken together, these studies not only provide evidence for the important role of GPER in the development of TAM resistance in TNBC cells, but also pinpoint a potential target therapy aimed at overcoming the resistance to this endocrine treatment.

### 4.3. Androgen Receptor (AR) and TAM Resistance

The androgen receptor (AR) is a member of the steroid hormone receptor superfamily, a class of receptors that function through their ability to regulate the transcription of specific genes. Furthermore, in women, adrenal and ovarian androgens are sources of pre- and post-menopausal estrogens, as these are converted into E2 [115]. Since it is not yet clear whether AR has a predominantly proliferative or anti-proliferative function, its biological role and significance as an independent predictor of clinical outcome in breast cancer remains controversial. However, an increasing set of data support a possible role of AR as a marker of prognosis in patients with ERα-positive breast cancer [116]. Approximately 90% of ERα-positive patients are also AR-positive (AR+) [117], and this is associated with favorable prognosis i.e., longer relapse-free survival, response to therapy, older age at diagnosis, lower tumor grade, lower Ki67 positivity and smaller tumor size [118,119,120].

#### 4.3.1. AR Signaling Pathway

**Figure 6:**
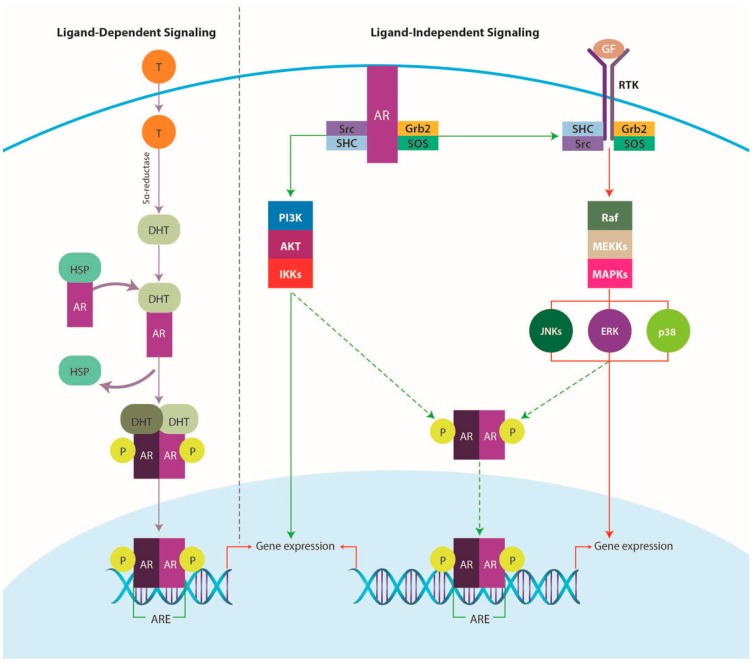
Androgen receptor (AR) signaling pathway. Ligand dependent and independent mechanisms. T: testosterone; DHT: 5-α dihydrotestosterone; HSP: heat shock protein; GF: growth factors; RTK: receptor tyrosine kinase.

The transcriptional activity of AR can be controlled by several pathways in order to transduce the AR signal and modulate AR-dependent transcription of target genes. The ability of AR to regulate gene transcription is through its interactions with specific DNA sequences located near or within the target gene promoter [121]. In this regard, AR can act as a ligand-dependent transcription factor via a classical genomic mechanism, which involves homodimerization and translocation to the nucleus upon binding the androgen hormones testosterone and 5-α dihydrotestosterone [122], interaction with AR response elements, and recruitment of co-regulators to elicit transcriptional changes [123]. Moreover, preclinical studies have also revealed a ligand-independent mechanism for triggering AR activation, through signaling pathways that include Janus kinase (JAK)/signal transducer and activator of transcription 3 (STAT3), MAPK, NOTCH and PI3K/mTOR/AKT [124,125]. Interestingly, some studies have indicated that AR can also cause rapid initiation of cytoplasmic signaling cascades, including activation of protein kinase A, protein kinase C and ERK, via a non-genomic mechanism involving binding cytoplasmic and membrane-bound proteins, such as c-Src [123] (Figure 6).

#### 4.3.2. AR and TAM Therapy

Although the role of AR in breast cancer is not fully clear, some studies have reported its implications in endocrine therapy response: while in ERα-positive tumors that respond to neoadjuvant endocrine therapy, the AR mRNA and protein expression decreases, in unresponsive tumors, the AR mRNA does not decrease. Moreover, increased AR expression has been observed in TAM-resistant breast cancer models in vitro and in vivo [126,127]. In fact, it has recently been reported that TAM-resistant tumors express higher levels of AR than TAM-sensitive tumors. These observations suggest that high AR expression may be detrimental for the outcome of TAM-treated ERα-positive breast cancer, as increased AR expression could potentially enhance the agonistic properties of TAM [126,127]. Interestingly, a recent study indicated that in ERα-positive breast cancers treated with TAM the ratio of nuclear AR to ER (AR:ER) rather than the level of AR expression may play a role in disease progression and response to treatment. In fact, women with tumors expressing a high AR:ER ratio (>2.0) had over four times higher risk for failure in TAM therapy compared to women with a low ratio (<2.0). Consequently, it was postulated that de novo or acquired resistance to anti-estrogen therapies could result from tumor cell adaptation, from estrogen dependence to androgen dependence [128,129]. These findings suggest that the nuclear AR:ER ratio may critically influence tumor biology and response to endocrine therapy, and that this ratio may be a new, independent predictor of response to traditional E2/ER-directed endocrine treatment [128].

### 4.4. Hedgehog(HH) Signaling Pathway and TAM Resistance

Components of the Hedgehog (HH) signaling pathway have recently emerged as a new molecular target in TAM-resistant breast tumors.

Ramaswamy et al. [130] were first to demonstrate that Glioma-associated oncogene homolog 1 (GLI1) mRNA, a marker of HH signaling activation, and its targets genes *SNAIL*, *BMI1* and *MYC*, were higher in TAM-resistant cells compared to TAM-sensitive MCF7 cells. Additionally, the MYC and BMI1 polycomb ring finger oncogene (BMI1) protein levels were directly correlated with increased TAM resistance. In the same study, serial passages of the resistant cells in mice, resulted in aggressive metastasic tumors with concomitant increases in the expression of markers of HH signaling and epithelial to mesenchymal transition. In a cohort of 315 patients with breast cancer, high GLI1 expression inversely correlated with disease-free and overall survival [130].

Matevossian and Resh [131] demonstrated that the HH acyltransferase (Hhat) is required for the proliferation of ERα-positive, HER2 positive and TAM-resistant breast cancer cells. Hhat is an enzyme catalyzing the N-terminal palmitoylation of Sonic HH (SHH), the major ligand of the pathway, a modification that is critical for SHH signaling activity [132,133]. Inhibition of Hhat by the small molecule RU-SKI 43, decreased anchorage-dependent and anchorage-independent proliferation of ERα-positive, but not triple negative breast cancer cells, and also reduced proliferation of HER2 amplified as well as TAM-resistant cells [131].

More recently, evidence was provided for a possible role of HH signaling pathway in breast cancer cells treated with TAM [134]. Using a panel of different breast cancer cell lines, we demonstrated that TAM modulates the expression of HH signaling components, including the terminal effector of the pathway, the transcription factor GLI1. Increased GLI1 gene expression and cellular proliferation following TAM treatment was observed in ERα-positive/HER2-negative and ERα-positive/HER2-positive cell lines. Activation of this pathway facilitates tumor growth and progression supporting an association of HH signaling with increased risk of metastasis and breast cancer-specific death [135]. These findings reveal that GLI1 activation can be implicated in the growth and progression of breast cancer, however, the precise mechanism by which GLI1 contributes to TAM resistance remains unclear.

#### 4.4.1. HH Signaling

The HH signal transduction cascade is a major pathway involved in embryonic development, cell proliferation, stem cell generation and tissue repair [136,137]. Deregulation of the HH signaling pathway is strongly correlated with various types of cancer, including basal cell carcinoma, medulloblastoma, rhabdomyosarcoma and tumors of the lung, breast, pancreas and prostate [138,139,140,141].

**Figure 7:**
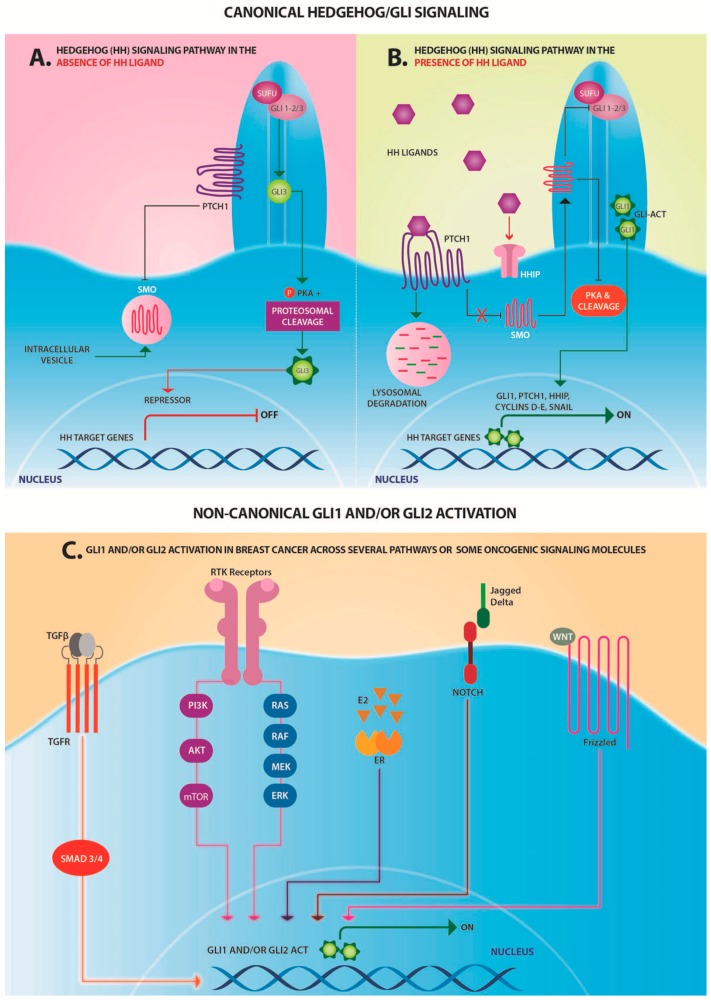
Simplified representation of canonical and non-canonical Hedgehog(HH) signaling. Canonical activation of the HH pathway. (**A**) In the absence of HH ligand, PTCH1 present in the primary cilium prevents Smoothened (SMO) trafficking and localization to the cilia. GLI mediators are in complex with proteins, including protein kinase A (PKA) and suppressor of fused homolog (SUFU), and generate repressor GLI forms, which translocate to the nucleus and inhibit transcription of HH signaling target genes; (**B**) In the presence of HH ligand, ligand binding to PTCH1 leads to PTCH1 internalization and degradation, SMO can traffic to the cilium and initiate a signaling cascade that processes the GLI proteins into transcriptional activator forms, which translocate to the nucleus and activate the expression of HH signaling target genes; (**C**) Activation of GLI1 and GLI2 in breast cancer by non-canonical pathways. Regulatory signaling cascades or regulatory proteins other than canonical Hedgehog signaling can modulate GLI1 and GLI2 expression, transcriptional activity and stability. Transforming growth factor β/transforming growth factor receptor (TGFβ/TGFR) signals can induce transcriptional up-regulation of GLI1 and GLI2 via SMAD. Receptor tyrosine kinases (RTK) signals via the PI3K-AKT signaling cascade can induce stabilization of the GLI1 protein. In ERα-positive breast cancer cells E2 induces expression of SHH and GLI1 independent of SMO activity. WNT signaling activation stimulates GLI2. GLI2 ACT, the activator form of GLI2.

The signaling cascade initiates at the transmembrane receptor Patched (PTCH), which interacts with HH ligands, e.g., sonic hedgehog (SHH), the most broadly expressed ligand [142], relieving its inhibitory effects on the signaling molecule Smoothened (SMO), another membrane-associated protein. The lack of PTCH repression allows SMO to initiate a series of intracellular events that culminate in the activation of the GLI (Glioma) family of zinc finger transcription factors, i.e., GLI1, GLI2 and GLI3 [143,144]. The GLI proteins can function both as activators and repressors, with GLI2 and GLI3 indeed possessing repressor and activator domains [145], whereas GLI1 acts only as an activator, since it lacks a repressor domain [146]. GLI1 is an oncogene and its increased expression is associated with many cancers, acting as a marker of HH signaling activation [147] (Figure 7).

The outcome of HH signaling varies depending on the cell type that responds to HH ligands, and may include expression of a variety of cell specific transcription factors mediating different developmental fate responses. Genes generally induced by HH signaling activity include *PTCH1* and *PTCH2*, *Hedgehog-interacting protein* (*HIP*) and *GLI1*, which can trigger positive or negative feedback on this pathway, modifying the strength or duration of the HH signal. Additional GLI targets include genes contributing to the regulation of proliferation and differentiation, e.g., *CCND1*, *CCND2*, *N-MYC*, *WNT*, *PDGFRA*, *IGF2*, *FOXM1* and *HES1* [146].

#### 4.4.2. HH Signaling in Breast Cancer

An increasing number of recent publications have highlighted a role for canonical and non-canonical HH signaling in breast cancer. Studies carried on the mammary gland have demonstrated the strict regulation of the HH pathway for normal development of this organ, since signaling de-regulation triggers embryonic and postnatal abnormalities [148,149]. It is therefore not surprising to find ample evidence for the involvement of this signaling pathway in breast cancer, as has recently been reviewed [149,150,151] (Figure 7).

SHH overexpression was documented to contribute to breast cancer development and progression in both ERα-positive and negative tumors. Kubo and colleagues found high expression of SHH, Patched 1 (PTCH1) and GLI1 in invasive carcinomas, in contrast to normal breast epithelia that do not express detectable levels of these proteins [152]. These data have been corroborated with additional findings in clinical samples and xenografts models [153]. O'Toole et al. reported that different subsets of breast cancer express HH ligands in the epithelium and/or stroma and also demonstrated that epithelial HH ligand expression is an early event in mammary carcinogenesis, strongly associated with a basal-like phenotype and poor outcome, in terms of metastasis and breast cancer-related death [135]. Jeng et al. found that high expression of PTCH1, GLI1 and SMO mRNA in breast cancer tissues correlates with invasiveness, and suggested that overexpression of these genes could be used as potential biomarkers for prediction of postoperative recurrence [154]. Recently, Noman et al. provided evidence for a relationship between high-level SHH expression and poor overall patient survival in TNBC [155]. Additionally, they suggested that the HH pathway in early stages of breast cancer enhances tumor growth and proliferation, while in later stages progression and recurrence. A possible role of hypomethylation of the SHH promoter, as a means to regulate SHH expression, has been put forward by Cui et al. [156].

Moreover, the role of the transcriptional effectors of HH signaling GLI1 and GLI2 has also been addressed. Nuclear GLI1 overexpression in breast cancer has been associated with increased invasiveness, early relapse after radical operation, and metastasis [152,157,158]. Furthermore, up-regulated GLI2 expression has been detected in progesterone receptor negative cases and correlated with increased Ki-67 proliferation index in invasive ductal carcinoma [159]. In an additional study, patients with increased GLI2 expression had a significantly lower overall survival [160]. Finally, it was demonstrated that GLI2 can mediate non-canonical activation of HH signaling in breast cancer [161].

#### 4.4.3. HH Signaling Crosstalk with Additional Pathways

Interactions between HH signaling and other pathways during normal development of the mammary gland and in breast cancer have frequently been reported [162,163,164,165]. A link between ERα and the HH pathway in human breast cancer was first highlighted by Koga et al. [163]. More recent data indicate that HH ligands in mammary epithelial cells can operate through both c-Src and ERα resulting in the activation of ERK1/2 [162].

An additional pathway that can interact with HH signaling in breast cancer is TGFβ, which induces transcriptional up-regulation of GLI1 and GLI2 [166,167]. Moreover, it has been shown that RTK signals, via the PI3K-AKT signaling cascade, can induce stabilization of the GLI1 protein [168]. Similar observations were reported by Ramaswamy et al., who hypothesized not only that the PI3K/AKT pathway is involved in HH signaling activation, but also that it may have a role in promoting TAM resistance [130]. Additionally, WNT signaling regulates GLI2 during development and is involved in modulating the expression and functionality of the GLI proteins in several malignancies, including breast cancer [153,169,170].

It has also been suggested that NF-κB, a key transcription factor that orchestrates numerous biological processes, including proliferation, apoptosis and inflammatory responses, is, at least in part, responsible for up-regulating SHH [156]. Finally, NOTCH receptor activation can also increase SHH expression through rapid modulation of cytoplasmic signals, including AKT and the mammalian target of rapamycin mTOR [171,172] (Figure 7).

### 4.5. Non-Coding RNAs and TAM Resistance

Non-coding RNAs (ncRNAs) are RNA transcripts that do not encode proteins. These include not only the functionally well-established tRNAs, rRNAs, and spliceosomal or snRNAs but also other types of ncRNAs, many of which are cell-type specific and involved in central biological processes [173,174]. Accumulating evidence indicates that most of the mammalian genome is transcribed and the resulting ncRNAs can actively regulate gene expression, exemplified by their role in the process of maturation, stabilization and/or degradation of protein coding mRNAs, and their enhancer/silencer impact on neighboring and distant genes [174,175].

These novel ncRNAs form two classes depending on their size, the short ncRNA (sncRNAs) and the long ncRNAs (lncRNAs) [176,177,178,179]. SncRNAs are RNA transcripts less than 200 nucleotides and include microRNAs (miRNAs), piwi-interacting RNAs (piRNAs), and small interfering siRNAs. In contrast, lncRNAs can range from 200 nucleotides to ~100 kilobases. Both classes of ncRNAs are recognized as key players in the pathogenesis of human cancer, with potential roles as biomarkers or therapeutic targets [180,181]. Expression of certain ncRNAs, and, more specifically, miRNAs and lncRNAs, has clearly been identified as biomarkers in the diagnosis, progression, prognosis, and response to treatment for certain cancers, acting either as tumor suppressors or oncogenes and, in some cases, as both [182].

#### 4.5.1. miRNAs

**Figure 8:**
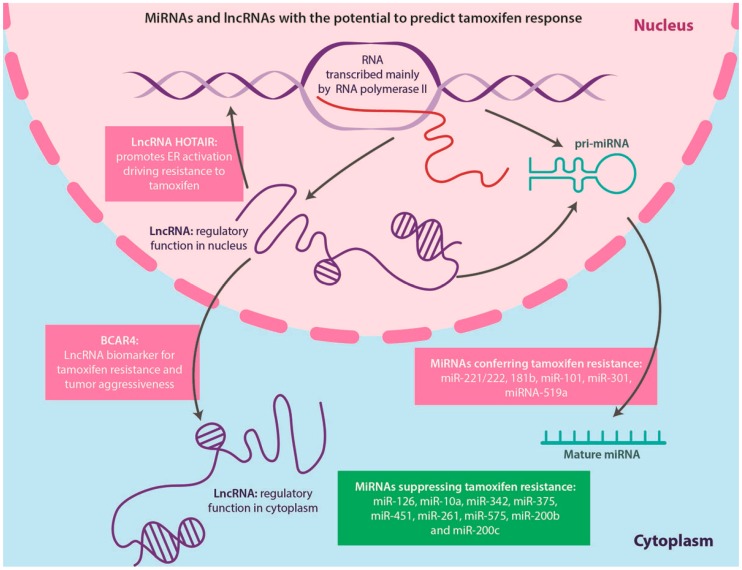
MicroRNAs (MiRNAs) and long non-coding RNAs (lncRNAs) with the potential to predict tamoxifen response. The majority of miRNA and lncRNAs are transcribed by RNA polymerase II. MiRNAs are frequently originating from introns of protein-coding transcripts but can also be processed from lncRNAs, via precursors called pri-miRNAs. In breast cancer, miRNAs have been suggested to be important for predicting tamoxifen response, since they can modulate the effects of anti-estrogen therapy. LncRNAs can function either in the nucleus or the cytoplasm and recent studies have demonstrated their role in endocrine resistance. For example, HOTAIR was shown to promote ER activation, driving resistance to tamoxifen and breast cancer anti-estrogen resistance 4 (BCAR4) was found to be a biomarker for tamoxifen resistance, with prognostic value for tumor aggressiveness.

miRNAs, first described in 1993, are small (22–24 nucleotides), single stranded non-coding, evolutionarily conserved RNA molecules [183,184], which are related to but distinct from siRNAs, and regulate mRNA translation and/or stability [185]. miRNAs are transcribed by RNA polymerase II and processed either from introns of protein-coding transcripts [186] or from lncRNAs (Figure 8), forming precursors called pri-miRNAs [187,188,189]. In cancer, miRNAs are deregulated and can act as tumor suppressors negatively affecting tumor growth or as oncogenes (termed oncomiRs), which are overexpressed in cancer and promote tumor formation [182,190]. miRNAs have been suggested to be important prognosticator markers in breast cancer and some currents studies aim to identify miRNAs with the potential to predict TAM response (Figure 8); however, their role in acquired endocrine-resistant breast cancer is not fully understood [191]. For example, overexpression of miR-221/222 confers resistance to TAM in MCF7 cells and correlates with HER2 positivity in primary human breast tumors [192,193]. Additionally, increased expression of miR-181b has been observed in TAM-resistant breast cancer [192,194], miR-101 promotes estrogen independent growth and causes TAM resistance in ERα-positive breast cancer cells [195], up-regulated miR-301 increases proliferation, migration, invasion, and tumor formation, and also has been associated with TAM resistance in MCF7 cells [196] and miRNA-519a confers TAM resistance in MCF7 cells through regulation of the cell cycle and apoptosis [197].

On the other hand, some miRNAs, which suppress TAM resistance in breast cancer, have also been identified. Hoppe et al. [198] demonstrated that miR-126 and miR-10a are markers for tumor recurrence in postmenopausal patients with early stage ERα-positive breast cancer treated with TAM and suggested that breast cancer metastasis may be favored by the loss of miR-126 expression [198]. The tumor suppressor role of miR-126 is further supported by the findings of decreased expression of this miRNA in highly metastatic MDA-MB231 breast cancer cell derivatives in mice [199]. miR-10a is downregulated in metastatic mouse mammary tumor cells; however, little is known whether a similar scenario is occurring in human breast cancer. It is speculated that the protective effect of miR-10a within the context of TAM treatment may be explained by maintaining the apoptotic capacity of tumor cells, since miR-10a directly targets *HOXA1*, which plays an oncogenic role in MCF10A cells via modulation of the anti-apoptotic factor BCL2 [198,200]. In addition, recent studies in ERα-positive cell lines have shown that restoring certain miRNAs sensitized these cells to TAM. Such miRNAs include miR-342 [201], miR-375 [202], miR-451 [203], miR-261, miR-575 [204], miR-200b and miR-200c [205,206] (Figure 8).

#### 4.5.2. LncRNAs

The majority of lncRNAs are the result of RNA polymerase II activity, and include 5’ capping and other transcriptional modifications, e.g., splicing and polyadenylation. Functional lncRNAs form stable secondary and tertiary structures, which confer unique biological properties, and may be found both in the nucleus or the cytoplasm [207]. Experimental evidence has indicated that lncRNAs contribute to a vast array of biological processes, including physiological as well as pathological conditions. Some studies have revealed that lncRNAs can display enhancer-like functions [208,209], with their mode of action ranging from modulation of protein expression to epigenetic transcriptional regulation and mRNA processing [189,210].

In breast cancer, lncRNAs have been demonstrated to play a leading role in initiation, progression and anti-estrogen resistance [211,212,213,214,215]. Moreover, a new molecular classification of breast cancer based on lncRNA expression has been put forward, and almost two thirds of the lncRNAs expressed in breast cancer were found localized at enhancer regions [216]. Additionally, lncRNA HOTAIR was highly upregulated in tumors of TAM resistant breast cancer patients compared to the primary tumors prior to treatment. These results provide evidence that HOTAIR significantly contributes to the proliferation of TAM resistant cells, suggesting that this lncRNA can promote ER activation in the absence of estrogen to drive TAM resistance [217].

LncRNA breast cancer anti-estrogen resistance 4 (BCAR4), normally found in human placenta and in oocytes, is present at high levels in breast tumors and associated with endocrine resistance and increased invasiveness. BCAR4 overexpression in TAM-sensitive cells blocked the anti-proliferative effects of TAM, likely via interactions with ERBB receptors inducing their phosphorylation [214,215,218] (Figure 8).

## 5. Clinical Use of TAM in Combination with Other Agents

Although TAM is widely used in the treatment of ERα-positive breast cancer patients, it has been reported that the use of AIs, e.g., anastrozole, letrozole and exemestane, as well as fulvestrant, offer better clinical outcomes by improving disease-free survival and reducing the risk of recurrence [10,35,219].

In addition to the development to endocrine resistance, another limitation of TAM is the toxicity associated with its use. Specifically, increases in endometrial cancer and thromboembolic events have limited the use of the drug by high-risk women, who would, otherwise, benefit from it. In order to overcome these adverse side effects, novel strategies have been proposed. Some of these approaches include the use of lower doses, which are anticipated to be associated with lower toxicity, the topical application of either TAM or its active metabolites and the use of combination therapies.

### 5.1. Lower Doses of TAM

A lot of research effort has focused on the use of lower doses of TAM, with these studies indicating that this approach minimizes toxicity without affecting the drug’s chemopreventive activity in the breast [220,221]. Data from animal studies indicate that a reduction in the TAM standard dosage of 20 mg/day to 1 mg/day does not diminish the drug’s inhibitory activity on mammary tumor formation [222] and does not affect a large number of biomarkers, most of which are surrogate markers of cardiovascular disease [223].

### 5.2. Topical Application of Either TAM or Its Active Metabolites

Another strategy to overcome the adverse effects of TAM is the topical application of either TAM or its active metabolite (4-OH-TAM) directly onto the breast. The purpose of this approach is to reduce the distribution of drug to tissues susceptible to TAM-induced toxicity. Rouanet and colleagues observed that the daily application of 1 or 2 mg of 4-OH-TAM hydroalcoholic gel on breast skin resulted in sufficient concentrations of the drug in the tissue to achieve inhibition of tumor cell proliferation to the same degree seen with the standard dose of oral TAM, but with much lower plasma levels [224]. Similar results were obtained in a phase II trial of Afimoxifene (4-OH-TAM gel) for cyclical mastalgia in premenopausal women [225]. In this study, the use of 4-OH-TAM gel (4 mg/day) delivered potent and sustained antiestrogenic effects to the target tissue, while avoiding the side effects associated with first-pass metabolism of TAM. The results of these studies indicate that the use of percutaneous 4-OH-TAM gel has a local impact on tumor proliferation, suggesting its possible use in future prospective trials of chemoprevention.

### 5.3. Use of Combination Therapies: GPER Inhibitors and TAM

Considering the recent evidence indicating that TAM can activate non-genomic GPER/ERK signaling and enhance breast cancer cell growth, inhibition of ER/GPER/ERK signaling could provide a new therapeutic option for TAM-resistant breast cancer cells. In this regard, several compounds that specifically inhibit GPER, including estriol and G15, have been described. Estriol binds to GPER and inhibits downstream signaling, while G15, a substituted dihydroquinoline, binds to GPER with high affinity and blocks calcium mobilization by E2 in breast cancer cells [113]. Moreover, it was recently shown that G15 improves the response of TAM-resistant xenografts to endocrine treatment [43]. These combination therapies could therefore restrain tumor progression, by increasing apoptosis, and restore the cytotoxic effects of TAM in drug-resistant cells [42,43].

### 5.4. Use of Combination Therapies: ARs Inhibitors and TAM

Cochrane et al. presented the first preclinical evidence indicating that inhibition of AR by enzalutamide may be an effective therapeutic strategy not only for ERα-negative/AR-positive but also for ERα-positive/AR-positive breast cancers. Additionally, high levels of AR relative to ER may also pinpoint a subset of breast cancers patients that would respond more favorably to enzalutamide alone or in combination with TAM or AIs [128]. Enzalutamide (formerly MDV3100) is an AR signaling inhibitor that binds AR with high affinity, impairing AR nuclear translocation [226,227] and decreasing ERα-mediated proliferation. The observed effect of enzatulamide in ERα-positive and ERα-negative breast cancers highlights the possible role for ARs in breast tumor growth.

### 5.5. Use of Combination Therapies: HH Inhibitors and TAM

Inactivation of the HH pathway can occur at various steps of the signaling cascade and several inhibitors have been developed and used in preclinical and clinical cancer trials.

The selective Hhat inhibitor RU-SKI 43, which blocks SHH formation, has proved to be an effective compound that reduces the proliferation of ERα-positive breast cancer cells [131]. SMO inhibition, via cyclopamine and its derivatives, prevents the downstream activation of the pathway and its transcriptional effectors, the GLI proteins [228]. Studies have revealed that blocking SMO with vismodegib can effectively reduce cellular proliferation [130,229] and inhibit the tumor growth of TAM-resistant xenografts in mice [130]. Vismodegib is a well-tolerated drug that offers an excellent outcome in canonical HH signaling-dependent cancers, e.g., basal cell carcinoma [228,230]. The limitation of SMO antagonists relates to the activation of the GLI factors independently of SMO in certain cancers [231]. In-line with this is the increasing focus on inhibitors that act at downstream steps of the pathway [139].

Interestingly, Fan et al. and Della Corte et al. reported that the antidiabetic drug metformin exerts anticancer effects through the inhibition of the HH signaling pathway in breast cancer cells, decreasing the expression levels of SHH, SMO, PTCH and GLI1 [232,233].

Taken together, the above set of data suggest that combinations of TAM with HH signaling inhibitors may be quite effective in overcoming endocrine resistance in breast cancer.

### 5.6. ncRNAs: Therapeutic Targets in TAM Resistance

Recent studies have focused on drugs targeting nucleic acids in order to develop breast cancer therapies, with the objectives centering on either inactivating ncRNAs, which confer resistance to TAM, or enhancing the effect of ncRNAs that restore susceptibility to anti-estrogen treatment [206,234,235]. These emerging therapies include ribozymes, siRNAs, antisense oligonucleotides (ASOs) or their chemically tailored analogs (known as locked nucleic acids, LNAs), which can alter RNA splicing and induce degradation of the targeted RNA via RNaseH. Such approaches can inhibit the production of an endogenous miRNA, while synthetic or miRNA mimics can be used to treat a deficiency in miRNA expression. Similar methodologies can also be employed to modulate the production of lncRNAs in order to enhance sensitivity to TAM [206,236,237].

## 6. Conclusions

Resistance to endocrine therapy is a significant clinical problem in breast cancer treatment. Consequently, efforts to dissect the molecular mechanisms that underlie the development of resistance are well justified. This analysis will also provide the means for the development of new therapeutic targets for breast cancer. The current evidence suggests that combination therapies and lower TAM doses should be applied in order to overcome endocrine therapy resistance and minimize the adverse effects of TAM, respectively.
